# Modelling the potential use of pre-exposure prophylaxis to reduce nosocomial SARS-CoV-2 transmission

**DOI:** 10.1371/journal.pcbi.1013361

**Published:** 2025-08-05

**Authors:** Lauren Stewart, Stephanie Evans, Teresa Brevini, Fotios Sampaziotis, Christopher J. R. Illingworth

**Affiliations:** 1 MRC University of Glasgow Centre for Virus Research, Glasgow, United Kingdom; 2 Fungal, HCAI, AMU & Sepsis Division, UK Health Security Agency, London, United Kingdom; 3 Statistics, Modelling and Economics, UK Health Security Agency, London, United Kingdom; 4 Cambridge Stem Cell Institute, Cambridge, United Kingdom; 5 Cambridge Liver Unit, Cambridge University Hospitals NHS Foundation Trust, Cambridge, United Kingdom; 6 Department of Medicine, University of Cambridge, Cambridge, United Kingdom; Northeastern University, UNITED STATES OF AMERICA

## Abstract

The nosocomial transmission of respiratory pathogens is an ongoing healthcare challenge, with consequences for the health of vulnerable individuals. Outbreaks in hospitals can require the closure of bays or entire wards, reducing hospital capacity and having a financial impact upon healthcare providers. Here we evaluate a novel strategy of pre-exposure prophylaxis as a means to reduce the nosocomial transmission of SARS-CoV-2. We model the effect of ursodeoxycholic acid (UDCA) upon levels of angiotensin-converting enzyme 2 (ACE2) expression, SARS-CoV-2 viral entry, and ultimately the probability of an infection. We then implement this model within simulations describing the spread of SARS-CoV-2 infections within a hospital context, simulating an intervention in which UDCA is given to patients on a ward for 10 days following the detection of a case of SARS-CoV-2 on that ward. Under default model parameters we infer a potential 17% reduction in the nosocomial transmission of SARS-CoV-2 to patients, with increased importation of cases into the hospital increasing the effectiveness of the intervention, and of the order 1000–2000 patient treatment days per nosocomial patient infection prevented. Our study provides preliminary evidence of the value of pre-exposure prophylaxis with UDCA as a strategy to reduce nosocomial SARS-CoV-2 transmission.

## Author summary

Preventing SARS-CoV-2 transmission is of particular importance in hospital environments. Individuals in hospital can be more vulnerable to infection. Dealing with outbreaks can consume limited hospital resources. Here we use mathematical modelling to assess the potential for a drug, UDCA, which has commonly been used in treating liver disease, to be used in a hospital to prevent SARS-CoV-2 infection. We model the process whereby UDCA reduces the expression of ACE2, a protein used by the SARS-CoV-2 virus to infect cells, estimating the effect of the drug in preventing viral transmission. We next model the effectiveness of the drug in preventing transmission in simulated hospital environments. Our work suggests that the use of UDCA following the detection of SARS-CoV-2 on a ward could reduce transmission to patients by approximately 17%, with this result dependent upon the length of time for which UDCA was given to patients, and the rate of importation of new SARS-CoV-2 cases into the hospital.

## Introduction

The SARS-CoV-2 virus has had a major impact upon human health [1,2]. As such, a key priority has been the identification of public health interventions which either prevent or reduce the impact of infection.

Vaccines have been shown to reduce the chances of infection leading to hospitalisation or death [3], albeit that the immunity gained from vaccination wanes over time [4], while virus evolution creates a need to continually re-evaluate vaccine effectiveness [5]. Other lines of defence against SARS-CoV-2 include antiviral drugs, such as pavloxid [6], remdesivir [7] and molnupiravir [8]. The US Food and Drug Administration has issued an emergency use authorisation for a monoclonal antibody therapy for pre-exposure prophylaxis [9]. These interventions have their own limitations, with for example a five-day course of molnupiravir being associated with a lower SARS-CoV-2 viral load five days after treatment, but a higher viral load after 14 days [10]. Non-pharmaceutical interventions, such as handwashing, masking, and social distancing, have helped to prevent infection [11].

Hospitals and care homes have been the focus of particular attention in the monitoring and prevention of SARS-CoV-2 transmission. Even as the consequences of COVID have attenuated for the general population [12], the concentration of potentially vulnerable individuals in these environments creates an enhanced need for action. One study early in the pandemic estimated that close to 15% of SARS-CoV-2 cases in hospital were the result of hospital-acquired infection [13]. Accordingly, studies have evaluated the use of mask-wearing by health care workers and patients in hospitals [14,15]. The installation of air cleaning units on a hospital ward was shown to reduce the concentration of airborne particulates of a size commensurate with airborne viral transmission [16]. Air cleaning has been associated with a 22% reduction in nosocomial transmission (95% CI 47% to -14%) [17].

A mathematical study identified pre-exposure prophylaxis (PrEP) as being potentially the most effective complement to vaccination in preventing SARS-CoV-2 infection [18]. Mathematical modelling provides a valuable insight into potential therapeutic approaches in advance of committing financial and clinical resources to a real-world trial. However, PrEP approaches to combating SARS-CoV-2 are not simply theoretical constructs. A Mendelian randomisation study of potentially druggable proteins identified the genes ACE2 and IFNAR2 as priority targets for intervention [19]. The former is a cell receptor which critically must be bound by SARS-CoV-2 Spike protein prior to cellular entry [20], while the latter participates in the host innate immune response [21] against the virus. Higher ACE2 expression has been associated with more severe clinical outcomes of SARS-CoV-2 infection [22].

The discovery that the farnesoid X receptor (FXR) regulates ACE2 expression provided a novel approach to controlling SARS-CoV-2 infection [23]. Ursodeoxycholic acid (UDCA) is a well-tolerated and off-patent drug which down-regulates FXR, leading to a reduction in ACE2 expression. A cohort study identified a significant reduction in the odds of developing SARS-CoV-2 infection among patients with cirrhosis who were treated with UDCA [24], finding also a reduced risk of disease severity among treated individuals. Although another study found no significant reduction in the risk of hospitalisation for COVID among treated individuals [25], the basic result has been replicated in independent studies [26,27], with high adherence to UDCA treatment [28], and an increased dose of UDCA [29] being associated with reduced rates of infection. A contrary result was reported in a smaller cohort of patients [30].

**Fig 1:**
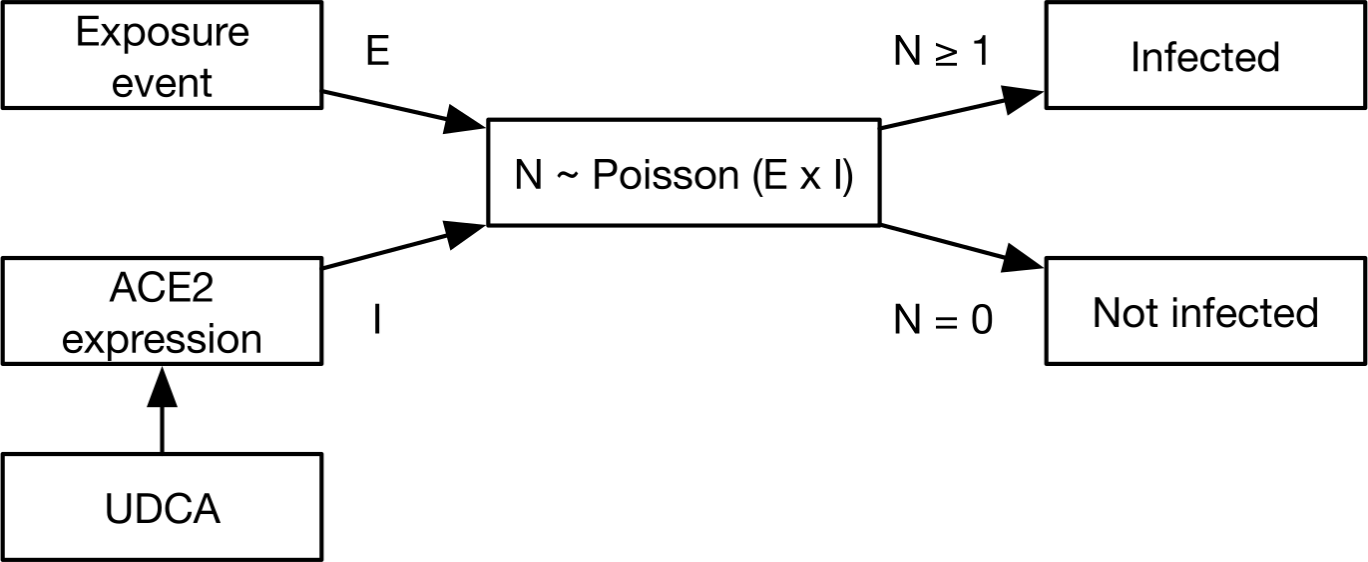
Schematic of our model of infection. An individual is exposed to SARS-CoV-2 in an event. The individual’s level of exposure is drawn from an underlying distribution, and represented by a parameter E. The probability that the individual is infected depends upon their level of ACE2 expression, represented by the parameter I. The value of I is expressed relative to the mean for an underlying population, so that the average untreated person has I = 1. The exposure event then leads to a stochastic outcome, whereby N viruses establish infection in the individual. If N is greater or equal to 1, the person is infected, but if N is zero the individual is not infected. Within this framework, UDCA acts to reduce ACE2 expression in a time-dependent way, with subsequent effect upon the probability of SARS-CoV-2 infection.

While clinical studies have compared individuals treated with UDCA to those not receiving the drug, there may exist a broader scope to use the drug in a responsive fashion, for example as pre-exposure prophylaxis given an anticipated period of greater risk of SARS-CoV-2 infection. In this study we use a two-stage mathematical model to evaluate the potential for UDCA to reduce nosocomial transmission. Firstly, we consider infection at the level of the individual, following an exposure to SARS-CoV-2. The chances of the individual being infected are altered by their level of ACE2 expression, with higher ACE2 expression increasing the probability of infection. UDCA reduces ACE2 expression, making infection less likely (Fig 1). Secondly, we integrate this model into a previously published simulation framework, which describes SARS-CoV-2 outbreaks in hospitals (S1 Fig). We evaluate how the use of UDCA on wards could potentially reduce the number of cases of nosocomial transmission to patients and health care workers (HCWs).

## Results

### Model parameterisation

**Fig 2:**
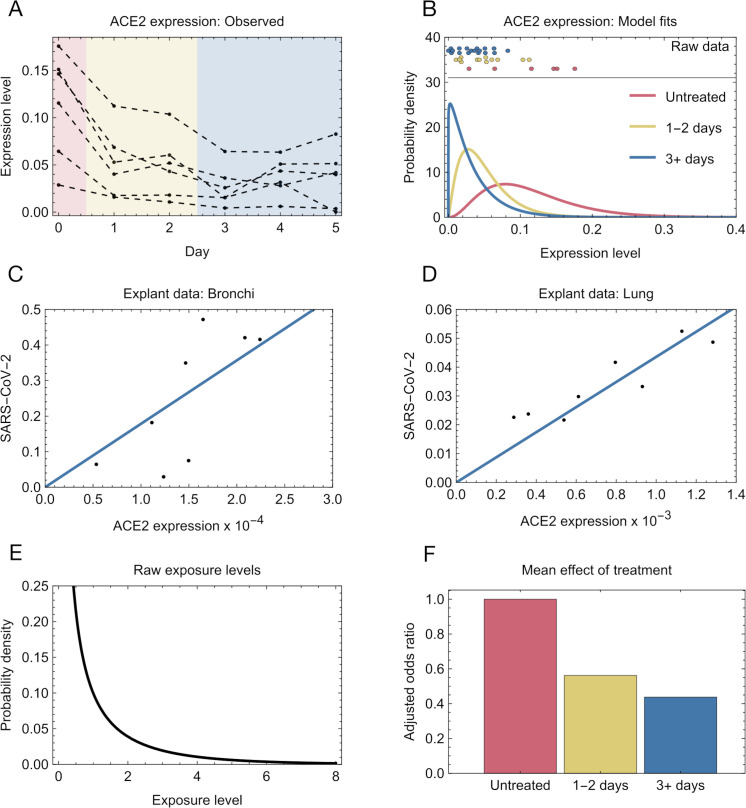
Fitting the individual model. **A.** qPCR measurements of the levels of ACE2 in nasal epithelial cells collected with nasopharyngeal swabs from six individuals who received 15 mg per kg per day of UDCA for five days, previously described by Brevini et al [23]. Shading indicates windows of time into which samples were divided for model fitting. **B.** Derived distributions of ACE2 expression levels in treated and untreated individuals. For simplicity we assumed that the ordering of individuals by ACE2 expression was preserved under treatment. **C.** Model fits to qPCR data describing levels of ACE2 expression and SARS-CoV-2 infection in a human explant. Data (black dots) describing samples collected from the bronchi were originally described by Brevini et al [23]. A linear model (blue) was fitted to the data. **D.** Linear model fit to qPCR data describing levels of ACE2 expression and SARS-CoV-2 infection in a lung samples. **E.** Distribution of SARS-CoV-2 exposure used in our model, fitted to published transmission bottleneck sizes [34] and a secondary attack rate [35]. An exposure of 1 means that an individual is infected by a Poisson random variable with parameter 1. If this random variable is equal to or greater than 1, the individual is infected, rather than not infected. **F.** Adjusted odds ratios of infection calculated across a population of individuals with a distribution of ACE2 levels.

We fitted distributions to data describing changes in ACE2 expression following treatment with UDCA (Fig 2A and 2B), changes in viral infection given differing levels of ACE2 expression using data from a lung explant study (Fig 2C and 2D), and levels of exposure to SARS-CoV-2 infection, based upon inferred numbers of viruses initiating infection (Fig 2E). Together, the derived models characterised the impact of UDCA treatment upon SARS-CoV-2 infection. A treated individual has a lower level of ACE2 expression, reducing the chances of infection given an exposure to SARS-CoV-2. Considering variation in baseline ACE2 levels across a population, we expect individuals who have been treated with UDCA for 1–2 days to be infected 56% as often as untreated individuals (Fig 2F). People who had been treated for 3 or more days were expected to be infected a mean 44% as often as untreated individuals.

### Hospital-based transmission

**Fig 3:**
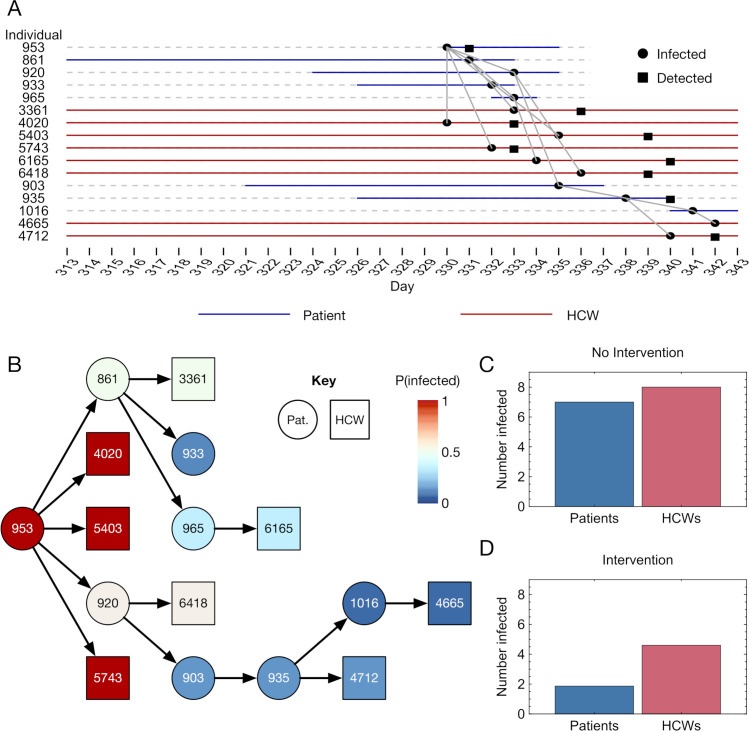
Application of UDCA treatment to a transmission network. **A.** Example case of nosocomial transmission within a hospital simulation. Day markers on the horizontal axis show time. Day 1 here is the first day of the simulation. Horizonal lines show times when individuals were in the hospital, coloured blue for patients or red for HCWs. Dashed grey lines are used as visual guides only, indicating times when individuals were not in the hospital. Black dots indicate dates on which individuals were infected, while squares indicate dates on which cases of infection were detected, where detection occurred. **B.** Representation of one realisation of the impact of UDCA treatment upon the transmission network. The ward upon which the outbreak began was an intervention ward at the time of patient 953 being introduced onto the ward. Colours show the sometimes reduced probabilities of individuals being infected with SARS-CoV-2 following the treatment of patients with UDCA. Reductions are stochastic, due to random sampling of the baseline ACE2 expression levels of individuals, and are cumulative, as non-infected individuals cannot transmit the virus. **C.** Numbers of secondary cases of SARS-CoV-2 infection in patients and HCWs in the network in the absence of an intervention. **D.** Numbers of secondary cases given treatment with UDCA.

To understand the potential effect of UDCA upon nosocomial transmission, simulations of hospital outbreaks were run, initially, to describe an environment in which UDCA was not used. The effect of treatment was represented by retrospective changes made to each simulation. Data from an outbreak extracted from one simulation shows how UDCA had the potential to cut short chains of transmission events (Fig 3A). In this case, an infected individual, with ID number 953, was introduced onto a ward, leading to a large outbreak involving both patients and healthcare workers.

In our simulated intervention, upon the detection of a case of SARS-CoV-2 on a ward, all patients on that ward, plus any arriving onto that ward during the intervention, were treated for 10 days with UDCA. The impact of UDCA was measured in terms of reduced probabilities of infection: Someone who was infected in the original simulation (which we denote as being infected with probability 1) was infected with probability p ≤ 1 in the case of the intervention. An example is shown in Fig 3B. Here, the ward onto which patient 953 was introduced was already marked as an intervention ward, so that patients in contact with patient 953, and those further down the network, were already treated with UDCA, reducing their chances of being infected. Reductions in transmission were compounded: Someone who was not infected could not infect others down the original transmission chain. Healthcare workers in the network were not treated, but acquired a lower risk of infection via a reduction in their exposure to infected patients. Across multiple transmissions within the network the probability of an individual being infected could be greatly reduced; for example the intervention results in individual 4665 having only a 7.5% chance of being infected. Within this network the numbers of secondary infections were reduced from 7 patients and 8 HCWs to a mean of 1.9 patients and 4.6 HCWs (Fig 3C and 3D).

**Fig 4:**
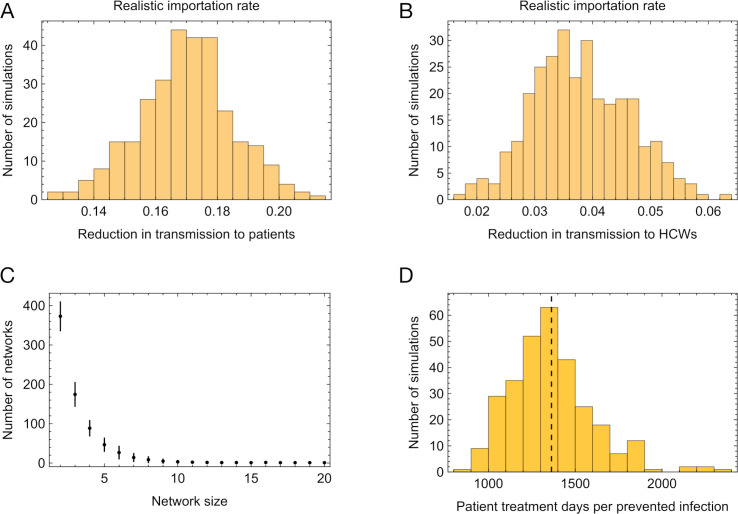
Reduction in transmission caused by the simulated use of UDCA. **A.** Reductions in nosocomial transmissions to patients, measured across five stochastic replicates of each of 60 hospital simulations. **B.** Reductions in nosocomial transmission events to healthcare workers, measured across five stochastic replicates of each of 60 hospital simulations. **C.** Variation in the reduction in nosocomial transmissions to patients, varying the length of the intervention, given the detection of a case upon a ward. **D.** Total length of interventions in a hospital according to the length of an individual intervention, measured in ward-days, across the 624 days of the simulation.

Applied to a set of simulated data, describing nosocomial transmission in 60 hospitals over a window of 610 days, our simulated intervention reduced cases of nosocomial transmission among patients by approximately 17%, with a 95% confidence interval representing differences between simulations of (14% - 20%) (Fig 4A). As modelled, the intervention reduced nosocomial infection of HCWs by close to 4% (Fig 4B). These figures are lower than obtained for the example network of Fig 2, which was an outlier in terms of its large size. Outbreaks in the simulated data were usually small. Most cases of infection in hospital did not lead to transmission, and the most common instance of nosocomial transmission involved one person infecting another, with no further transmission (Fig 4C). Including cases of non-transmission, the mean transmission network involved 0.8 cases of nosocomial transmission: An analysis of these networks suggested that the average case of infection led to 0.44 transmission events (S2 Fig), consistent with range of 0.04 to 0.81 calculated in a paper describing hospital transmission in 2020 [31]. Under default model conditions, an average of just over 1360 patient days of treatment was associated with each avoided case of nosocomial transmission to a patient (Fig 4D).

Our results were sensitive to the parameters of our model. Considering our initial fit of a model to ACE2 expression data, we derived distributions within the bounds of uncertainty for which the difference between untreated and treated (day 3+) individuals was either minimised or maximised (S3 Fig). Minimising the impact of ACE2 reduced the effect of the intervention on patients from a mean of 17% to a mean 11% reduction in cases, and a mean of 2192 patient days of treatment per avoided cases of patient infection. Increasing the impact of ACE2 increased the effect of the intervention to a mean reduction of 21% in patient cases, equivalent to 1079 patient days of treatment per avoided case of infection.

**Fig 5:**
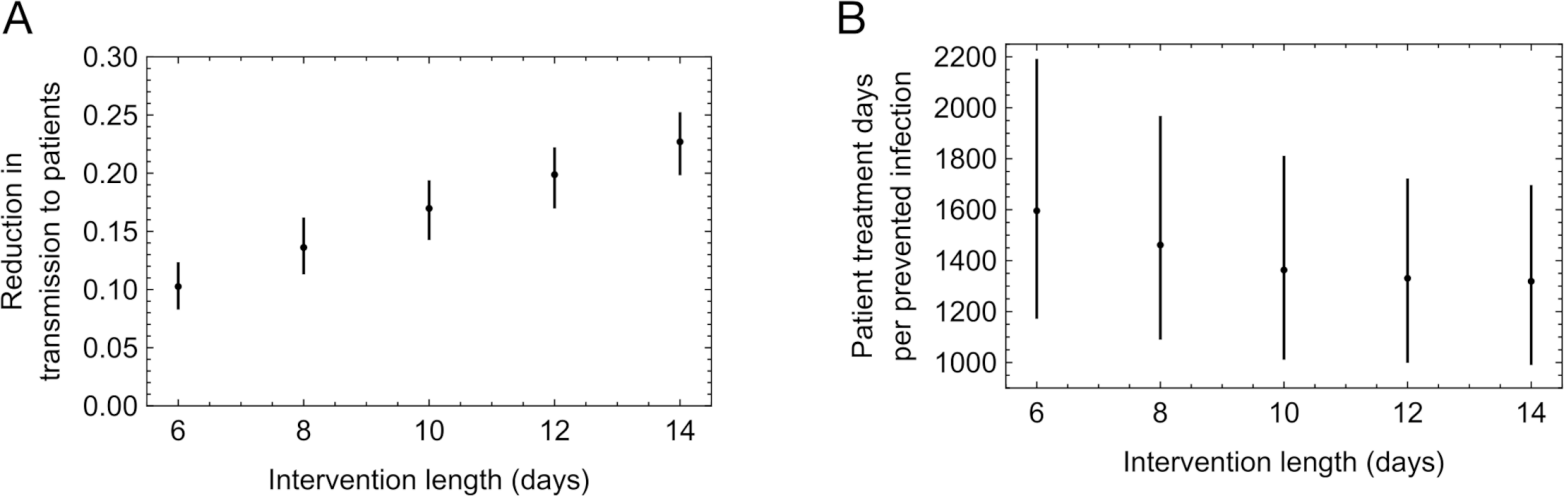
Effect of altering the intervention length upon the effectiveness and efficiency of the intervention. **A.** Changes in the proportion of patient cases of nosocomial transmission prevented by the intervention as a function of intervention length. Black dots show mean reductions, with lines spanning a 90% confidence interval. **B.** Changes in the number of patient treatment days per prevented patient infection. Black dots show mean numbers of days, with lines spanning a 90% confidence interval.

We further explored changes in the window of intervention following the detection of a case of infection. A longer window of intervention both reduces the probability that an outbreak is over before the intervention is complete, via cases in which there is a long period of time between symptom onset and transmission, and also prevents transmission from unrelated outbreaks, in the event that a patient with SARS-CoV-2 is moved onto a ward where there has previously been an intervention. The time-dependent effect of UDCA means that it is particularly effective in a case where treatment is started prior to an outbreak occurring, as when an outbreak begins on a ward which has already been marked out for intervention. As such, increasing or decreasing the length of the intervention in our model had a corresponding effect on the proportion of patient cases of nosocomial transmission avoided (Fig 5A), from a mean of 10% given a 6-day window of treatment to a mean of 23% given a 14-day window of treatment. Further, longer windows of treatment increased the efficiency of the intervention, with reductions in the mean numbers of patient days of treatment per prevented case of infection falling from 1596 to 1319 as the intervention length was increased from 6 to 14 days (Fig 5B).

**Fig 6:**
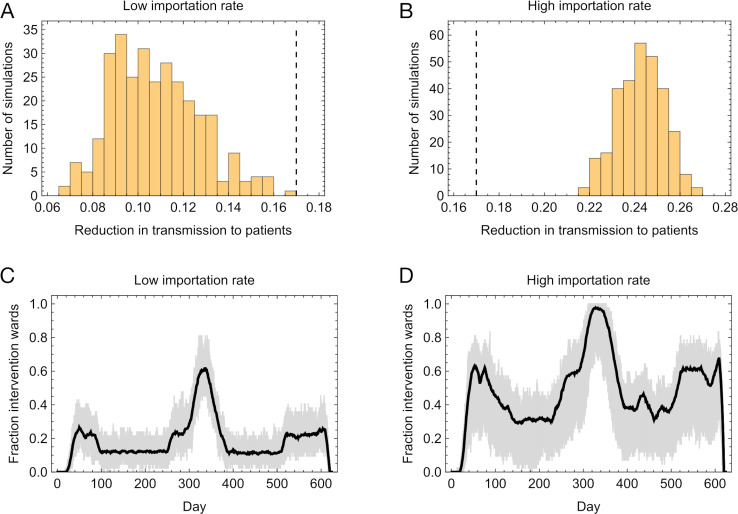
Effect of variation in the rate of importation of SARS-CoV-2 cases to the hospital. **A**. Reductions in nosocomial transmissions to patients, calculated under a low importation rate. The vertical black dashed line shows the mean of this statistic under a realistic importation rate, modelling importations in the UK between 2020 and 2021. **B.** Reductions in nosocomial transmissions to patients, calculated under a low importation rate. The vertical black dashed line shows the mean of this statistic under a realistic importation rate. **C.** Fraction of wards in which the intervention was taking place, plotted by time. The black line shows the mean calculated across 60 simulations with a low importation rate. The gray shaded region shows the range across simulations. **D.** Fraction of wards in which the intervention was taking place, plotted by time. The black line shows the mean calculated across 60 simulations with a high importation rate. The gray shaded region shows the range across simulations.

The effect of the intervention was also sensitive to the simulated level of community infection, which in our model determines the rate of importation of SARS-CoV-2 patients into a hospital. Halving the importation rate compared to the default led to an estimated 11% reduction in nosocomial cases in patients, while doubling the rate of importation relative to default led to an estimated 24% reduction in nosocomial cases (Fig 6A and 6B). Although a higher importation rate led to more cases of SARS-CoV-2 in hospital, and an increased number of cases of nosocomial transmission, the distribution of the sizes of transmission networks was not substantially altered (S4 Fig). Rather, the difference in outcome arose from a higher number of transmission networks occurring on wards that had previously been marked out for intervention. Where we modelled a high rate of importation, a situation arises in which nearly all hospital wards are included in the intervention (Fig 6C and 6D). At this point any new cases of nosocomial transmission occurring in the hospital involve already-treated patients.

**Fig 7:**
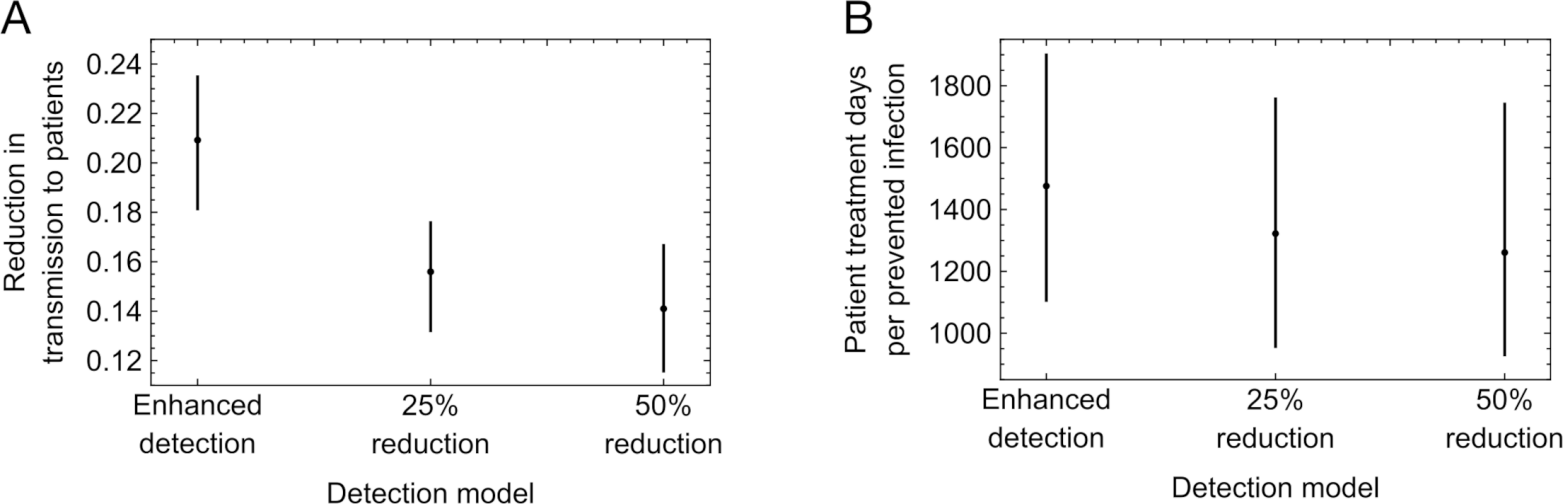
Performance of the intervention under enhanced and reduced detection models. The enhanced model was as the original simulation, but with the modification that all symptomatic cases of patient infection were instantaneously detected. The reduced models were as the original simulation, but with 25% and 50% of detected cases marked as undetected. **A.** Reduction in cases of nosocomial transmission in patients given different models of detection. **B.**

Our simulations describe by default a detection regime that matches hospital practice during the earlier phases of the pandemic, with symptomatic cases being PCR tested within 2 days, and symptomatic arrivals into hospital being tested straight away (S1 Text). Within our simulations there was a distribution of times from the day that a cluster of cases was detected to the time an infection in the cluster occurred (S5 Fig). To explore the extent to which our results were altered by changes in testing, we reran calculations with improved detection, such that all symptomatic cases were instantaneously detected, and with worsened case detection, with the hospital simulations being edited to that 25% or 50% of cases that originally were detected were marked as undetected. Improved detection led to a larger reduction in patient cases of nosocomial transmission, with a mean of 21% of patient cases of nosocomial transmission prevented, while worse detection led to smaller reductions, with means of 16% and 14% of patient cases prevented given the removal of 25% or 50% of detections (Fig 7A). Surprisingly the intervention was more efficient at lower rates of case detection, with 1483 patient days of treatment per case avoided given the higher rate of detection, but 1326 and 1267 patient days of treatment per case avoided given the removal of 25% or 50% of detections (Fig 7B). We attribute this result to the focusing of interventions towards larger outbreaks where detection is poorer. Given a reduction in testing, large clusters of cases are disproportionately likely still to be detected, so that the intervention is focused upon these clusters. By contrast, where testing is enhanced, new interventions are disproportionately focused upon cases where less transmission took place.

## Discussion

We have here considered the use of a potential candidate for use as pre-exposure prophylaxis against SARS-CoV-2 infection. We begin with a model of SARS-CoV-2 infection and ACE2 expression, considering changes in ACE2 expression in response to UDCA treatment, the consequences of those changes for virus entry into cells, and the likely levels of exposure to the virus encountered by individuals: This model produces a result consistent with observational studies. Applying the model next to simulated data describing a hospital setting, our approach suggests that a proposed intervention using the drug might lead to a reduction of around 17% in nosocomial transmission to patients. Accounting for various uncertainties in the model suggested that, implemented in the way we describe, in the region of 1000–2000 patient days of treatment would be required to prevent each case of nosocomial transmission. Our work provides an indicative and preliminary assessment of the potential for UDCA as to be used for pre-exposure prophylaxis to combat nosocomial transmission.

Our modelling provides some insight into the potential value of pre-exposure prophylaxis in a real-world setting. Where modelling has suggested the use of pre-exposure prophylaxis as an effective tool to prevent SARS-CoV-2 infection, the implicit need for action to be taken prior to an exposure limits its value. In modelling a hospital outbreak we have chosen a setting in which the potential risk of infection is unusually high, in which cases of infection are being actively monitored, and in which treatment could be administered by competent medical professionals. In this setting, the intervention had both direct and indirect effects, reducing transmission in the outbreak that triggered the intervention, but further reducing transmission in any independent outbreaks that by chance occur on a ward in which the intervention is already in force. The drug is more efficient in this later context: The delay between starting treatment and achieving reduced ACE2 expression means that outbreaks on wards of already-treated patients are reduced by the greatest margin.

Our work highlights a distinction between preparatory and responsive approaches to reducing infectious disease transmission. Preparatory approaches, such as improving ventilation, mask-wearing, and hand hygiene, create an environment in which transmission is intrinsically less likely. Responsive approaches, which are implemented following the detection of an outbreak, are limited by imperfect surveillance, and in the case of UDCA, by transmission events having occurred prior to the intervention taking effect. Our estimated reduction in transmission of 17% compares to a recent study suggesting a 22% reduction in transmission with the use of improved ventilation [17], but may be optimistic.

Our model has broader implications going beyond nosocomial transmission, suggesting that people with higher baseline levels of ACE2 expression are more likely to be infected with SARS-CoV-2. A higher ACE2 expression level implies that viruses to which a person is exposed are more likely to gain entry into cells and initiate infection. Our model also implies that a high baseline ACE2 expression level will reduce the efficacy of UDCA in preventing infection. S6 Fig describes expected reductions in transmission for high and low baseline levels of ACE2 expression.

Hospital environments are complex and our modelling approach is built upon multiple assumptions. Due to data availability our model of exposure to SARS-Cov-2 was constructed using data describing transmission in a domestic, rather than a hospital setting. The simulated hospital environment, while representing the state of the art, also contains multiple simplifications. For example, the effect of UDCA was evaluated in a post-hoc manner, running a hospital simulation, identifying transmission networks, then considering a counterfactual scenario in which UDCA was used in an intervention. While enabling simple statistical comparison between intervention and non-intervention cases, this in other ways complicates our results. For example, a healthcare worker who avoided being infected due to the indirect effect of UDCA would retain a risk of being infected at some later date via the local community. Our post-hoc approach neglected this risk, as a HCW whose infection was prevented by UDCA in our analysis would stay uninfected for the remainder of the simulation. Our post-hoc approach would in other ways underestimate the effect of UDCA. Where patients were multiply exposed to SARS-CoV-2, UDCA could potentially slow down outbreaks on hospital wards, with transmission events occurring later in time. The delayed impact of UDCA on ACE2 levels means that later transmission events are more likely to be prevented, but these timing effects were neglected.

Other simplifications applied. Our model assumes that the distribution of ACE2 levels in the hospital population mirror those of the general population. Further, simulations of hospitals simplify elements such as the movement of patients and staff between wards which likely impact upon transmission. Differences between hospital environments, such as those of hospital architecture, are not captured by our model. The relationship in our model between UDCA treatment and ACE2 expression was based on data from only six patients, making this one of the key areas of uncertainty in our model. Given the different results obtained in clinical studies of UDCA, this aspect of the data would benefit from further research. However, our model did give a result consistent with that described in one cohort study. Previously, John et al found an adjusted odds ratio for risk of symptomatic COVID infection of 0.54 (confidence interval 0.39 – 0.73) among individuals with cirrhosis who were treated with UDCA [23]. Considering an individual who had been treated for at least 3 days prior to exposure to SARS-CoV-2, our model suggested that continuous treatment with UDCA would reduce the incidence of COVID infection by 56%, equivalent to an adjusted odds ratio of 0.44.

The intervention we propose is described in a simplified fashion. Although long-term therapy with UDCA is well-tolerated [32], aspects such as negative drug-drug interactions may prevent the giving of the drug in a universal manner to a cohort of patients. We have here considered the use of UDCA for pre-exposure prophylaxis in a situation where full clinical supervision is possible. Other realistic intervention strategies might use the drug only in subsets of cases where SARS-CoV-2 infection would have particularly severe consequences, such as in a care home or with more vulnerable patients. The authors do not recommend the use of UDCA in any situation except under the explicit guidance of a qualified physician.

## Methods

We built a model to estimate the effect of treatment with UDCA upon SARS-CoV-2 transmission. Our model has three parts, the first modelling the effect of UDCA upon ACE2 expression in an individual, the second modelling the effect of changing ACE2 expression upon virus entry into cells, and the third modelling the likely distribution of exposure to SARS-CoV-2 viruses, given knowledge of transmission bottlenecks from cases of SARS-CoV-2 infection.

### Changes in ACE2 expression given treatment with UDCA

Given data from a set of individuals describing variation in ACE2 levels after commencing treatment with UDCA [23], we used a time-dependent gamma distribution *G* to represent the effects of the drug. In this equation *x* describes ACE2 expression level. The FindMaximum routine in the Mathematica software package was used to identify optimal parameters a and b within this model.


$$G(a,b,t,x)=P\left(ACE2=x|a,b,t\right)=\frac{x^{a(t)-1}{b(t)}^{-a(t)}e^{-x/b(t)}}{\Gamma \left(a(t)\right)}$$ (1)

G(a,b,t) was here characterised using data describing three discrete time intervals, for *t* = 0 days, *t* ∈ {1, 2} days, and *t* ≥ 3 days. As such, we inferred three values for each of the distribution parameters *a*(*t*) and *b*(*t*), one corresponding to each of those time periods. Raw data are shown in Fig 2A; the inferred distributions are shown in Fig 2B.

### Modelling of the relationship between ACE2 expression and viral infection

Data describing ACE2 expression and SARS-CoV-2 infection levels from different cell types within a lung explant was used for modelling purposes [23]. A linear model describing the relationship between ACE2 receptor availability, *[ACE2]*, and the amount of SARS-CoV-2 viruses getting into cells, *V*, provided a qualitatively good fit to these data (Fig 2C and 2D).


V=a[ACE2] (2)

Our use of a linear model for this relationship greatly simplifies our overall, integrated model. Further investigation suggests that it provides an acceptable approximation to what is likely a more complex picture of glycoprotein-receptor dynamics in the host (S2 Text and S1 Table).

### Inference of a distribution of levels of exposure to virus

We generated a model of the number of viruses expected to initiate infection during SARS-CoV-2 transmission. Given a level of exposure to viruses *E*, we modelled the transmission bottleneck size *N*_*b*_ (i.e., the number of viruses initiating an infection [33]) as being Poisson distributed with parameter *E*.


$$P\left(N_{b}=n|E\right)=\frac{E^{n}e^{-E}}{n!}$$ (3)

We assumed that exposure can be described by a Gamma distribution, such that E obeys the formula


$$P\left(E=x|\alpha ,\beta \right)=\frac{x^{\alpha -1}\beta^{-\alpha }e^{-x/\beta }}{\Gamma (\alpha )}$$ (4)

Where Γ(*α*) describes the gamma function


$$\Gamma (\alpha )=\int_{0}^{\infty }t^{\alpha -1}e^{-t}dt$$ (5)

We next inferred parameters α and β for this model using data from two publications describing household SARS-CoV-2 transmission. A study of SARS-CoV-2 transmission in households inferred bottleneck sizes for 20 cases of infection [34]. We took these sizes as being indicative of viral transmission events. Next, we used the result from a meta-analysis of household transmission studies, of a secondary attack rate for SARS-CoV-2 of 18.9% [35], to estimate that for each 20 cases of infection, there were an additional 83 cases of exposure not leading to infection. We thus compiled a dataset of 103 outcomes of exposure, 20 of which matched the bottlenecks inferred by Lythgoe et al [34], and 83 of which involved zero viruses. Data describing inferred bottlenecks for these 20 cases is shown in S7 Fig.

For a given set of parameters *α* and *β*, we represented the gamma distribution by a discrete set of 999 equally spaced quantiles *E*_*i*_(*α*, *β*). We then calculated the mean likelihood of observing each bottleneck *n*_*j*_ in the dataset, summing these over the 103 different bottlenecks.


$$\log L\left(\alpha ,\beta |\left\{n_{j}\right\}\right)=\sum_{j=1}^{103}\frac{1}{999}\sum_{i=1}^{999}\log P\left(N_{b}=n_{j}|E_{i}(\alpha ,\beta )\right)$$ (6)

Values of α and β which maximised this likelihood were found using the Mathematica software package. We inferred the values *α* = 0.1558 and *β* = 2.797. The inferred distribution of exposure values is shown in Fig 2E.

### Combined model of viral transmission in the presence or absence of treatment with UDCA

Under the assumption of a linear relationship between ACE2 expression and viral infection, we derived a model of viral transmission in treated and untreated individuals in a heterogeneous population. In our model, a person *p* receives a stochastic exposure *e* to viruses characterised by a random draw from the Gamma distribution *E*(*α*, *β*).


e~E(α,β) (7)

This exposure was scaled in a linear fashion by the individual-specific term *I*(*r*, *t*), which describes the relative ACE2 expression of the person. Here the parameter r was uniformly sampled from the interval (0,1); for example *r* = 0.12 would imply that *I*(*r*, *t*) = *x* was equal to the 12^th^ centile of the distribution *G*(*a*, *b*, *t*, *x*). In this manner, the effective exposure of a person *p* who had undergone *t* days of treatment with UDCA, was described as


$$E_{p}=\frac{I(r,t)}{I\overset{―}{\left(r,0\right)}}e$$ (8)

where the denominator represents the mean level of ACE2 expression for an untreated individual. The number of viruses initiating infection in this person was then given by the Poisson random variable


$$P\left(N_{b}=n|E_{p}\right)=\frac{E_{p}^{n}e^{-E_{p}}}{n!}$$ (9)

In this model, infection occurred if *N*_*b*_ was greater or equal to 1, and did not occur if *N*_*b*_ was equal to zero. Basic statistics of infection were calculated across distributions of ACE2 expression for treated and untreated individuals, integrating over a representative range of levels of exposure.

### Simulation of hospital transmission events

We used a published individual-based model of nosocomial transmission to simulate SARS-CoV-2 in hospitals, including the importation of cases from the community, the occasional spread of infection between patients and health care workers (HCWs) in hospitals, and the detection or non-detection of these cases [36,37]. Within this modelling framework simulations were conducted describing high, realistic, and low levels of importation of cases into hospitals. ‘Realistic’ levels were derived from now-casted community levels of infection for the East of England, spanning a window of 600 days during the SARS-CoV-2 pandemic [38]. High and low levels represented 2-fold and 0.5 fold changes to these values. For each level of importation, twenty sets of parameters representing rates of transmission to and from healthcare workers and patients were generated, consistent with evidence from a study of transmission in hospitals [39]. Three statistical replicates were generated for each set of parameters, making a total of 180 simulations. Model parameters and their origins are described in S2 Table.

For each simulation, transmission networks were identified, comprising sets of patients and HCWs who infected each other in hospital, alongside the dates of these transmission events. We modelled an intervention in which, upon the detection of a case of SARS-CoV-2, all patients on the ward in which the case was detected were given a 10-day course of treatment with UDCA. Where new patients arrived on the ward within 10 days of the commencement of the intervention, these patients were also started on a 10-day course of UDCA treatment. We then retrospectively altered the identified transmission networks. Transmission events in these networks were assigned a probability of occurrence equal to one. These probabilities were then altered according to UDCA treatment, with a treated patient having a reduced probability of being infected. Alterations in probabilities were then evaluated in a compound manner. For example, if in a simulation person A infected person B, who infected person C, treating person B would reduce the probability of person C being infected, even if C was untreated. The expected reduction in the number of cases of SARS-CoV-2 nosocomial infection was therefore calculated.

Our model of nosocomial transmission accounted for the variation in baseline susceptibility to SARS-CoV-2 infection implied by our model of infection and treatment. To achieve this we generated a distribution of the baseline ACE2 expression of an individual conditional upon their having been infected with SARS-CoV-2. This distribution differs from the gamma distribution fitted to the untreated ACE2 expression levels: The fact of having been infected increases the probability of an individual having a high level of baseline ACE2 expression.

To calculate the conditional distribution of ACE2 we calculated quantiles of the distributions of baseline ACE2 expression *G*(*a*, *b*, 0, *x*), and of the exposure distribution *E*(*α*, *β*)(*y*), calculating the products of these values. We then identified probabilities of infection for each datapoint, normalising these to sum to one


$$P(x,y)=\frac{1-e^{-G(a,b,0,x)E(\alpha ,\beta )(y)}}{\sum_{x,y}1-e^{-G(a,b,0,x)E(\alpha ,\beta )(y)}}$$ (10)

These values were then normalised to sum to one across all *x* and *y*, before summing across exposures *y* to calculate a probability distribution from which values of ACE2 expression could be sampled. In evaluating nosocomial transmission, individuals who were infected in the initial simulation were assigned baseline levels of ACE2 expression from this discrete distribution before evaluating the individual-specific effect of UDCA upon their probability of having been infected. The discrete distribution and the inferred probability density function are shown in S8 Fig.

The assignment of baseline ACE2 expression levels to individuals in the simulation creates a stochastic element to the outcome of the intervention. Five replicate calculations were performed for each run of the hospital simulation code to capture statistical variation from this source.

### Estimate of an R number from hospital simulation data

From simulations of hospital transmission in which the intervention was not performed, we identified clusters of transmission, recording the sizes of these clusters. Clusters of size one, where an infected person did not transmit SARS-CoV-2, were included in thi0.s analysis. Where we identified *c*_*n*_ clusters of size *n*, we calculated a formula based upon the Borel distribution


$$L(R)=\sum_{n}\left[c_{n}\log\left(\frac{{nR}^{n-1}e^{-nR}}{n!}\right)\right]$$ (11)

finding a value R which maximised this expression.

## Supporting information

S1 Text Overview of the method for simulating outbreaks in hospitals. (DOCX)

S2 Text Comparison of linear and sigmoidal models for modelling the relationship between ACE2 expression and SARS-CoV-2 infection. (DOCX)

S1 Table BIC scores for linear and sigmoidal models describing the relationship between ACE2 expression and SARS-CoV-2 infection in lung and bronchi cells from a human explant. (XLSX)

S2 Table Parameter values used in the simulation of nosocomial transmission. (DOCX)

S1 Fig Schematic overview of our model of outbreaks in hospital. Individuals are described using an SEIR model (Susecptible, Exposed, Infected, Recovered). Patients infect each other directly via transmission (P2P direct), each other indirectly via contamination of the environment (P2P indirect), and healthcare workers (P2H). Healthcare workers (HCWs) are infected in the community independent of the hospital, and infect patients (H2P), and each other in hospital (H2H).
(PDF)

S2 Fig Nominal estimate of an R value for transmission in our hospital model. This calculation was performed on identified clusters of transmission between individuals from the original model, using the sizes of clusters to infer a value. We note that transmission is limited by the nature of the hospital environment, and by individuals not remaining in hospital for the duration of their infection. In this sense the estimate cannot be compared directly to estimates of R_0_ for general SARS-CoV-2 transmission.
(PDF)

S3 Fig Uncertainty in models of ACE2 expression in untreated and treated individuals. Solid lines show the original fits of a model to data describing ACE2 expression levels. Dashed lines show low (reduced mean expression) and high (increased mean expression) fits, differing by one log likelihood unit from the best fitting model. Data are shown. A. For untreated individuals. B. For treated individuals. A model in which the effect of UDCA was reduced was constructed by combining the low mean untreated case with the high mean 3 + days case. Likewise a model in which the effect of UDCA was increased was constructed by combining the high mean untreated case with the low mean 3 + days case.
(PDF)

S4 Fig Network size numbers and distribution for different simulated importation rates. A cluster size of 2 indicates one person infecting another, with no further transmission.
(PDF)

S5 Fig Statistics of infection and detection from simulations carried out under the default testing model. **A.** Distribution of days of infection of cases in clusters relative to the day on which the cluster was detected. The vertical dashed line shows the mean value: the mean date of infection was 0.51 days before the detection of the first case in a cluster. Data are shown for detected clusters. **B.** Infection times for undetected clusters of infection relative to the first time of infection within a cluster. The vertical dashed line shows the mean value: the mean date of infection was 0.29 days after the first infection in the cluster.
(PDF)

S6 Fig Expected distributions of SARS-CoV-2 transmission bottlenecks. Inferred values are shown for among individuals at the 10th and 90th centiles of ACE2 expression, in untreated individuals, and in individuals treated for more than three days with UDCA. In both cases UDCA reduces expected bottleneck sizes, but with a more dramatic reduction in the probability of transmission (i.e., bottleneck size ≥ 1) among individuals with low ACE2 expression.
(PDF)

S7 Fig Data describing SARS-CoV-2 transmission bottlenecks, from an earlier publication [34]. Data describe the number of SARS-CoV-2 viruses initiating infection in 20 cases of household transmission. In our study these data were augmented with cases describing non-infection, that is with transmission bottleneck zero, reflecting a published secondary attack rate for SARS-CoV-2 in a domestic context [35]. Our basic exposure model, described in Fig 1A, was then fitted to the augmented data.
(PDF)

S8 Fig Distribution of baseline ACE2 expression conditional upon an individual having been infected. **A.** Probabilities of discrete values of baseline ACE2 expression. **B.** Probability density function implied by the discrete distribution.
(PDF)
