# Supplementary material for: Modelling the potential use of pre-exposure prophylaxis to reduce nosocomial SARS-CoV-2 transmission
Source: PLoS Comput Biol. 2025 Aug 5;21(8):e1013361. doi: 10.1371/journal.pcbi.1013361 (PMC12370187; doi:10.1371/journal.pcbi.1013361)
Supplement: S1 Text — (DOCX) [file pcbi.1013361.s001.docx]

**S1 Text: Modelling the potential use of pre-exposure prophylaxis to reduce nosocomial SARS-CoV-2 transmission**

**Individual-based model methodology**

The individual-based model (IBM) used in this study has been previously published and a full model description has been provided [1]. Here we provide a high-level description of the model with points that are relevant for the present study.

The IBM captures transmission between patients in a single hospital. The model is parameterised to simulate observed patient and healthcare worker infection data from across the COVID-19 pandemic. Admissions rates and length of stays are fitted to hospital-episode statistics (HES) and adjusted for sex and infection status. Transmission rates are parameterised to produce realistic numbers of observed infections in line with national data on nosocomial patient infections from the pandemic period and HCW infection data from the SARS-CoV-2 immunity and reinfection evaluation (SIREN) study [2]. The proportion of infections that were patient-to-patient, patient-to-HCW etc were fitted to published genomic data from two periods in the pandemic [3]. An example parameter set is given in Appendix 1 below. In this study we simulate 60 distinct parameter sets that all produce outcomes that fit to the calibration data where each parameter set represents a hospital with slightly different dynamics (e.g. parameter set 1 has a slightly higher HCW-to-patient transmission rate and parameter set 2 has a slightly higher patient-to-patient transmission rate but both parameter sets result in a similar number of overall patient infections).

The simulated hospital has 42 wards each containing four bays of six beds and patients are cohorted by symptom status on admission, with suspected or confirmed COVID-19 patients being placed into wards together and symptomatic patients on separate wards to COVID-19 patients. Once a patient has been assigned a bed they do not move throughout their hospital stay. There are 8000 HCWs in the model that work 12hr shifts. 30% of these HCWs are ward-based and work on the same ward with the same staff every shift with the rest being transient and visiting randomly selected patients at each timestep while at work. Patients can be infected by other patients on the same bay or ward (with different probabilities), and also by HCWs when being treated. HCWs can be infected by other HCWs that they come into contact with or by patients they are treating. HCWs that are not on shift can also be infected in the community at a rate proportional to the community prevalence. Patients that develop symptoms are PCR tested within 2 days of becoming symptomatic or on admission if they are admitted with symptoms. HCWs are assumed to test with lateral-flow devices and isolate for 7 days following the development of symptoms. The probability of detection of both tests was based on viral load and modelled using the approach presented by Quilty et al [4].

1. Evans S, Naylor NR, Fowler T, Hopkins S, Robotham J. The effectiveness and efficiency of asymptomatic SARS-CoV-2 testing strategies for patient and healthcare workers within acute NHS hospitals during an omicron-like period. BMC Infectious Diseases. 2024;24: 64. doi:10.1186/s12879-023-08948-9

2. Hall VJ, Foulkes S, Charlett A, Atti A, Monk EJM, Simmons R, et al. SARS-CoV-2 infection rates of antibody-positive compared with antibody-negative health-care workers in England: a large, multicentre, prospective cohort study (SIREN). Lancet. 2021;397: 1459–1469. doi:10.1016/S0140-6736(21)00675-9

3. Lindsey BB, Villabona-Arenas ChJ, Campbell F, Keeley AJ, Parker MD, Shah DR, et al. Characterising within-hospital SARS-CoV-2 transmission events using epidemiological and viral genomic data across two pandemic waves. Nature Communications. 2022;13: 671. doi:10.1038/s41467-022-28291-y

4. Quilty BJ, Clifford S, Hellewell J, Russell TW, Kucharski AJ, Flasche S, et al. Quarantine and testing strategies in contact tracing for SARS-CoV-2: a modelling study. Lancet Public Health. 2021;6: e175–e183. doi:10.1016/S2468-2667(20)30308-X
