# Supplementary material for: Modelling the potential use of pre-exposure prophylaxis to reduce nosocomial SARS-CoV-2 transmission
Source: PLoS Comput Biol. 2025 Aug 5;21(8):e1013361. doi: 10.1371/journal.pcbi.1013361 (PMC12370187; doi:10.1371/journal.pcbi.1013361)
Supplement: S2 Text — (DOCX) [file pcbi.1013361.s002.docx]

**S2 Text: Considering the use of a linear approximation to describe the relationship between ACE2 expression and the viral infection of cells.**

In our model we used a linear approximation to describe the relationship between ACE2 expression and the viral infection of cells, fitting models independently to data from lung and bronchi cells taken from a human explant.

The linear model facilitates a simple integration of the relationship between ACE2 expression and the extent of viral infection into the broader model. As doubling the expression leads to twice as many viruses gaining entry into a cell, this step becomes very straightforward. However, under a model of receptor association, the relationship between ACE2 receptor availability and the amount of SARS-CoV-2 viruses getting into cells, V, is expected to follow the Hill equation[1]. This equation, describing a sigmoidal curve would need more effort to integrate into our broader model for example requiring knowledge of the absolute level of ACE2 expression in the people in hospital we were modelling. At high ACE2 levels, close to saturation, a change in ACE2 expression might have little effect on the extent of viral infection.

To explore this relationship further, we carried out a formal model comparison, fitting two models to data collected from a lung explant experiment.

Linear model:

|  | $V=a\left[ ACE2 \right]$ | ( 1 ) |
| --- | --- | --- |

Hill equation:

|  | $V=\frac{m{[ACE2]}^{a}}{{c+[ACE2]}^{a}}$ | ( 2 ) |
| --- | --- | --- |

Models were fitted to the data using the NonlinearModelFit routine within the Mathematica software package, inferring different parameters for the lung and bronchi data; these data were not directly comparable due to different cell compositions of the different samples. Model fits were compared using the Bayesian Information Criterion (BIC)[2]. A comparison of BIC values identified a difference of 2.81 units in favour of the Hill equation model (S2 Table), suggesting positive support, but not strong support, for the more complex model[3].

We conclude that while the Hill equation provides a statistically better fit to the data, the approximation provided by the linear model is not unreasonably bad: Within physiological levels of ACE2 expression the large gains in tractability from the use of this model are not accompanied by a devastating loss of model performance.

**References**

1. Goutelle S, Maurin M, Rougier F, Barbaut X, Bourguignon L, Ducher M, et al. The Hill equation: a review of its capabilities in pharmacological modelling. Fundamemntal Clinical Pharma. 2008;22: 633–648. doi:10.1111/j.1472-8206.2008.00633.x

2. Schwarz G. Estimating the dimension of a model. Ann Statist. 1978;6: 461–464.

3. Raferty A. Bayesian Model Selection in Social Research. Sociological Methodology. 25: 111–163.
