# Supplementary material for: Modelling the potential use of pre-exposure prophylaxis to reduce nosocomial SARS-CoV-2 transmission
Source: PLoS Comput Biol. 2025 Aug 5;21(8):e1013361. doi: 10.1371/journal.pcbi.1013361 (PMC12370187; doi:10.1371/journal.pcbi.1013361)
Supplement: S2 Table — (DOCX) [file pcbi.1013361.s004.docx]

S2 Table: Parameter values used in the modelling of nosocomial transmission in hospitals and their origins

SUS is the NHS Secondary Uses Service repository for data.

| **General** |  |  |  |
| --- | --- | --- | --- |
| occupancy | Proportion of occupied beds in trust | 0.85 | Mean from individual trust data in SUS |
| hcws | Number of HCWs | 8000 | Mean from individual trust data |
| patients | Number of patients initially in trust | 860 | At 86% capacity[5] |
| testSens_PCR | Sensitivity of PCR test (false negative rate) | 0.95 | Woloshin et al.[6] |
| testSpec_PCR | Specificity of PCR test (false positive rate) | 0.995 | Woloshin et al.[6] |
| testSens_LFD | Sensitivity of LFD test (false negative rate) | 0.78 | PHE/University of Oxford [7] |
| testSpec_LFD | Specificity of LFD test (false positive rate) | 0.995 | PHE/University of Oxford[7] |
| incubationPeriodDays_shape | Shape variable for Gamma distribution for incubation period | 13.3 | He et al.[8] |
| incubationPeriodDays_rate | Rate variable for Gamma distribution for incubation period | 4.16 | He et al.[8] |
| onsetPeriodDays_mean | Mean for Log-normal distribution for onset period | 1.434065 | SPI-M communication |
| onsetPeriodDays_sd | SD for Log-normal distribution for onset period | 0.6612 | SPI-M communication |
| wards | Number of wards | 42 |  |
| shared_bays | Number of bays | 4 | ~80% beds are in shared rooms[9] |
| bedsPerBay | Number of beds in a shared room | 6 |  |
| **Patients** |  |  |  |
| probMale | Probability of patients being Male | 0.576 | SUS |
| ageShape | Shape component of Gamma distribution used to set age of patients on admission | 4.145 | Fit to data from SUS. Distribution of form Weibull(α,γ) where α = constant and γ = β_0_ + β_1_.Age+ β_2._Gender |
| ageScale | Scale component of Gamma distribution used to set age of patients on admission | 75.48 | Fit to data from SUS. Distribution of form Weibull(α,γ) where α = constant and γ = β_0_ + β_1_.Age+ β_2._Gender |
| shapeLOS_Susc | Shape parameter for Weibull distribution for used to set the LOS susceptible patients on admission | -0.1780082 | Fit to data from SUS. Distribution of form Weibull(α,γ) where α = constant and γ = β_0_ + β_1_.Age+ β_2._Gender |
| scaleLOS_Susc_int | Intercept for scale parameter for Weibull distribution used to set LOS for susceptible patients on admission | 2.331949 | Fit to data from SUS. Distribution of form Weibull(α,γ) where α = constant and γ = β_0_ + β_1_.Age+ β_2._Gender |
| scaleLOS_Susc_age | Age component of scale parameter for Weibull distribution used to set LOS for susceptible patients | -0.00546131 | Fit to data from SUS. Distribution of form Weibull(α,γ) where α = constant and γ = β_0_ + β_1_.Age+ β_2._Gender |
| scaleLOS_Susc_female | Female gender component of scale parameter for Weibull distribution used to set LOS for susceptible patients on admission | -1.242609 | Fit to data from SUS |
| scaleLOS_Susc_age_female | Interaction of age and female gender component of scale parameter for LOS distribution Weibull distribution for susceptible patients on admission | 0.01424664 | Fit to data from SUS. Distribution of form Weibull(α,γ) where α = constant and γ = β_0_ + β_1_.Age+ β_2._Gender |
| shapeLOS_Inf | Shape parameter for Weibull distribution used to set LOS for symptomatically infected patients on admission or following transmission in nosocomial cases | 0.07853536 | Fit to data from SUS. Distribution of form Weibull(α,γ) where α = constant and γ = β_0_ + β_1_.Age+ β_2._Gender |
| scaleLOS_Inf_int | Intercept for scale parameter for Weibull distribution used to set LOS for symptomatically infected patients on admission or following transmission in nosocomial cases | 2.595361 | Fit to data from SUS. Distribution of form Weibull(α,γ) where α = constant and γ = β_0_ + β_1_.Age+ β_2._Gender |
| scaleLOS_Inf_age | Age component of scale parameter for Weibull distribution used to set LOS for symptomatically infected patients on admission or following transmission in nosocomial cases | 0.002301093 | Fit to data from SUS. Distribution of form Weibull(α,γ) where α = constant and γ = β_0_ + β_1_.Age+ β_2._Gender |
| scaleLOS_Inf_female | Female gender component of scale parameter for Weibull distribution used to set LOS for symptomatically infected patients on admission or following transmission in nosocomial cases | -0.5465226 | Fit to data from SUS. Distribution of form Weibull(α,γ) where α = constant and γ = β_0_ + β_1_.Age+ β_2._Gender |
| scaleLOS_Inf_age_female | Interaction of age and female gender component of scale parameter for Weibull distribution used to set LOS for symptomatically infected patients on admission or following transmission in nosocomial cases | 0.006978023 | Fit to data from SUS. Distribution of form Weibull(α,γ) where α = constant and γ = β_0_ + β_1_.Age+ β_2._Gender |
| patientAsymProb | Probability a patient infection is asymptomatic | 0.4 | SUS |
| bP2P | Transmission rate to other patients |  | Calibrated |
| bH2P | Transmission rate from HCW to patients |  | Calibrated |
| bP2P_hosp | Indirect transmission rate from patients to other patients |  | Calibrated |
| recoveryRatePat_Shape | Shape parameter of Gamma distribution of recovery time distribution per day set at time of infection. | 1.43 | Fit to data from SUS |
| recoveryRatePat_Rate | Rate parameter of Gamma distribution of recovery distribution per day set at time of infection. | 0.542 | Fit to data from SUS |
| deathOnDisch_Inf_Int | Intercept of linear model for probability of death on discharge for infected patients, set on admission | -0.00043572 | Fit to data from SUS. Equation of form ax^2^ + bx + c |
| deathOnDisch_Inf_X | X component of linear model for probability of death on discharge for infected patients, set on admission | -0.00159802 | Fit to data from SUS. Equation of form ax^2^ + bx + c |
| deathOnDisch_Inf_X2 | X^2^ component of linear model for probability of death on discharge for infected patients, set on admission | 7.30529E-05 | Fit to data from SUS. Equation of form ax^2^ + bx + c |
| deathOnDisch_Susc_Int | Intercept of linear model for probability of death on discharge for non-symptomatic/uninfected patients, set on admission | -7.96613 | Fit to data from Cohen et al. [10] Equation of form ax^2^ + bx + c |
| deathOnDisch_Susc_X | X component of linear model for probability of death on discharge for non-symptomatic/uninfected patients, set on admission | 0.00102 | Fit to data from Cohen et al. [10] Equation of form ax^2^ + bx + c |
| deathOnDisch_Susc_X2 | X^2^ component of linear model for probability of death on discharge for non-symptomatic/uninfected patients, set on admission | 0.000896 | Fit to data from Cohen et al. [10] Equation of form ax^2^ + bx + c |
| testOnAdmProb_Inf | Probability a symptomatically infected patient will be tested on admission, set on admission | 0.95 | Estimated from individual NHSE trust data |
| testOnAdmProb_Other | Probability non-infected patient will be tested at random on admission | 0.05 | Estimated from individual NHSE trust data |
| testInHospProb | Probability a symptomatically infected patient infected nosocomially will be tested per timestep | 0.1 | Assumption that all patients will be tested within 2 days of developing symptoms |
| testPeriodSteps | Timesteps from test to result | 8 | Estimated from individual NHSE trust data |
| readmitProb | Probability a patient that develops symptoms after discharge will be readmitted within 14 days | 0.2 | Estimated from individual NHSE trust data |
| expOnAdmissionProb | Probability a susceptible patient will be exposed on admission (to be multiplied by the number of cases admitted that are known symptomatic) | 0.002631579 |  |
| retestProb_per_step | Probability a patient will be retested after day 5 (per step) | 0 | No retesting in this analysis |
| hcw_to_patient_contacts | Number of unique patients seen by a HCW every day (used to calculate exposure risk to COVID+ patients) | 20 | Cohen et al.[11] |
| allAdmTestStartDay | Day to start testing all admissions | 0 | Assume all admissions are always tested/retested |
| d3TestStartDay | Day to start retesting on day 3 | 0 |  |
| d5TestStartDay | Day to start retesting on day5 | 0 |  |
| non_covid_sympt_prob | Probability a non-infected patient with have COVID-19-like symptoms on admission | 0.1 | Assumption based on ILI proportions in a normal year[12] |
| d0_exp_prob | Proportion of new admissions that have previously recovered from COVID-19 | 0.5 | SUS |
| d0_exp_prob_omicron | Proportion of new admissions that have previously recovered from omicron | 0.05 | SUS |
| **HCWs** |  |  |  |
| shiftLengthHrs | Length of shift in hours (this is converted to timesteps within the model code) | 12 | Assumption |
| hcwAsymProb | Probability a HCW that becomes infected is asymptomatic | 0.4 | Assumption |
| bP2H | Transmission probability from patients to HCWs per timestep | 0.0000025 | Calibrated |
| bH2H | Transmission probability from HCWs to other HCWs per timestep | 0.0000001 | Calibrated |
| absentThroughSick_self | Probability a HCW will self-isolate per timestep | 0.01 | 34% over total infected time[13] |
| testProbHCWDays | Periodicity of HCW LFD testing (days) | 3.5 | NHSE LFD protocol[14] |
| lfd_test_compliance | Proportion of HCWs adhering to LFD testing regime | 0.7 | Expert opinion |
| absentDays | Number of days to be off work following a positive test | 7 |  |
| beginTestingDay | Day that LFD testing of HCW began | 253 |  |
| commScale | Scale of community acquisition rate for HCWs | 0.052 | CoMIX[15] |
| patient_to_hcw_contacts | Number of HCWs seen by a patient per day (for scaling transmission risk) | 18 | Cohen et al.[11] |
| prob_ward_based | Probability a HCW is based on a ward instead of moving around the hospital | 0.25 | Estimate from proportion of nursing staff out of all staff |
| days_to_vaccine_efficacy | Day after vaccination that protection begins | 21 |  |
| Vaccine_waning_per_step | Rate of vaccine waning per time step | 0.003 | Andrews et al [16] |
| Vaccine_efficacy_one_dose | Efficacy of one vaccine dose | 0.7 | SIREN study estimates[2] |
| Vaccine_efficacy_two_doses | Efficacy of two vaccine doses | 0.8 | UKHSA technical briefing |
| Vaccine_efficacy_one_dose_omicron | Efficacy of one vaccine doses against omicron | 0 |  |
| Vaccine_efficacy_two_doses_omicron | Efficacy of two vaccine doses against omicron | 0.32 |  |
| Vaccine_efficacy_three_doses_omicron | Efficacy of three vaccine doses against omicron | 0.62 |  |
| Vaccine_efficacy_no_doses_omicron_prior | Efficacy of protection against omicron in those that have prior infection | 0.44 |  |
| Vaccine_efficacy_one_dose_omicron_prior | Efficacy of one vaccine doses against omicron in those that have prior infection | 0.44 |  |
| Vaccine_efficacy_two_doses_omicron_prior | Efficacy of two vaccine doses against omicron in those that have prior infection | 0.6 |  |
| Vaccine_efficacy_three_doses_omicron_prior | Efficacy of three vaccine doses against omicron in those that have prior infection in those that have prior infection | 0.71 |  |
| hcw_vaccination_start_day | Day that vaccination program in HCWs commences | 281 | 8 December 2021 |
| **Comm** |  |  |  |
| careHomeProb | Probability a patient is from a care home | 0.02 | Health foundation communication |
| caseScale | Scaling factor for admissions rate | 1 |  |
| vacc_scale_from | Scaling factor for transmissibility from vaccinated individual | 0.5 | Harris et al. [17] |
| vacc_scale_to1 | Scaling factor for transmissibility to individual vaccinated with one dose (trans = 1-value) | 0.7 | Hall et al. [2] |
| vacc_scale_to2 | Scaling factor for transmissibility to individual vaccinated with two doses (trans = 1-value) | 0.7 | Andrews et al.[16] |
| var_scale_alpha | Scaling factor of transmission parameter for Alpha variant (compared to WT) | 1.56 | PHE technical report |
| var_scale_delta | Scaling factor of transmission parameter for Delta variant (compared to WT) | 1.99 | PHE technical report |
| var_scale_omicron | Scaling factor of transmission parameter for Omicron variant (compared to WT) | 2.32 | [18] |

1. Evans S, Naylor NR, Fowler T, Hopkins S, Robotham J. The effectiveness and efficiency of asymptomatic SARS-CoV-2 testing strategies for patient and healthcare workers within acute NHS hospitals during an omicron-like period. BMC Infectious Diseases. 2024 Jan 8;24(1):64.

2. Hall VJ, Foulkes S, Charlett A, Atti A, Monk EJM, Simmons R, et al. SARS-CoV-2 infection rates of antibody-positive compared with antibody-negative health-care workers in England: a large, multicentre, prospective cohort study (SIREN). Lancet. 2021 17;397(10283):1459–69.

3. Lindsey BB, Villabona-Arenas ChJ, Campbell F, Keeley AJ, Parker MD, Shah DR, et al. Characterising within-hospital SARS-CoV-2 transmission events using epidemiological and viral genomic data across two pandemic waves. Nature Communications. 2022 Feb 3;13(1):671.

4. Quilty BJ, Clifford S, Hellewell J, Russell TW, Kucharski AJ, Flasche S, et al. Quarantine and testing strategies in contact tracing for SARS-CoV-2: a modelling study. Lancet Public Health. 2021;6(3):e175–83.

5. NHS England. Statistics » Bed Availability and Occupancy Data [Internet]. [cited 2020 Feb 17]. Available from: https://www.england.nhs.uk/statistics/statistical-work-areas/bed-availability-and-occupancy/bed-data-day-only/

6. Woloshin S, Patel N, Kesselheim A. False Negative Tests for SARS-CoV-2 Infection - Challenges and Implications [Internet]. Vol. 383, The New England journal of medicine. N Engl J Med; 2020 [cited 2020 Sep 16]. Available from: https://pubmed.ncbi.nlm.nih.gov/32502334/

7. Preliminary report from the Joint PHE Porton Down & University of Oxford SARS-CoV-2 test development and validation cell: Rapid evaluation of Lateral Flow Viral Antigen detection devices (LFDs) for mass community testing [Internet]. [cited 2022 Jan 6]. Available from: https://www.ox.ac.uk/sites/files/oxford/media_wysiwyg/UK%20evaluation_PHE%20Porton%20Down%20%20University%20of%20Oxford_final.pdf

8. He X, Lau EHY, Wu P, Deng X, Wang J, Hao X, et al. Temporal dynamics in viral shedding and transmissibility of COVID-19. Nature Medicine. 2020 May 1;26(5):672–5.

9. AMR local indicators - produced by the UKHSA | Fingertips | Department of Health and Social Care [Internet]. [cited 2025 Apr 8]. Available from: https://fingertips.phe.org.uk/amr-local-indicators#page/3/gid/1938132917/pat/158/par/TE_trust/ati/118/are/RR8/iid/92084/age/-1/sex/-1/cat/-1/ctp/-1/yrr/1/cid/4/tbm/1

10. Cohen J, Bilsen J, Addington-Hall j, Löfmark R, Miccinesi M, S K, et al. Population-based Study of Dying in Hospital in Six European Countries [Internet]. Palliative medicine. 2008 [cited 2020 Jun 10]. Available from: https://pubmed.ncbi.nlm.nih.gov/18715968/

11. Cohen B, Hyman S, Rosenberg L, Larson E. Frequency of Patient Contact with Health Care Personnel and Visitors: Implications for Infection Prevention. Jt Comm J Qual Patient Saf. 2012 Dec;38(12):560–5.

12. UK flu levels according to PHE statistics: 2018 to 2019 [Internet]. GOV.UK. [cited 2025 Apr 8]. Available from: https://www.gov.uk/government/news/uk-flu-levels-according-to-phe-statistics-2018-to-2019

13. Kluytmans M, Buiting A, Pas S, Bentvelsen R, Bijllaardt W van den, Oudheusden A van, et al. SARS-CoV-2 infection in 86 healthcare workers in two Dutch hospitals in March 2020. medRxiv. 2020 Mar 31;2020.03.23.20041913.

14. Coronavirus » Novel coronavirus (COVID-19) standard operating procedure [Internet]. [cited 2020 Dec 1]. Available from: https://www.england.nhs.uk/coronavirus/wp-content/uploads/sites/52/2020/11/C0873_i_SOP_LFD-rollout-for-asymptomatic-staff-testing_phase-2-trusts-v1.1_16-nov20.pdf

15. Jarvis C, Coletti P, Backer J, Munday J, Faes C, Beutels P, et al. Social contact patterns following the COVID-19 pandemic: a snapshot of post-pandemic behaviour from the CoMix study. 2023.

16. Andrews N, Stowe J, Kirsebom F, Toffa S, Rickeard T, Gallagher E, et al. Covid-19 Vaccine Effectiveness against the Omicron (B.1.1.529) Variant. N Engl J Med. 2022 21;386(16):1532–46.

17. Harris M, Clark J, Coote N, Fletcher P, Harnden A, McKean M, et al. British Thoracic Society guidelines for the management of community acquired pneumonia in children: update 2011. Thorax. 2011 Oct 1;66(Suppl 2):ii1–23.

18. Sofonea MT, Roquebert B, Foulongne V, Verdurme L, Trombert-Paolantoni S, Roussel M, et al. From Delta to Omicron: analysing the SARS-CoV-2 epidemic in France using variant-specific screening tests (September 1 to December 18, 2021) [Internet]. medRxiv; 2022 [cited 2025 Apr 8]. p. 2021.12.31.21268583. Available from: https://www.medrxiv.org/content/10.1101/2021.12.31.21268583v1
