# Supplementary material for: Modelling the potential use of pre-exposure prophylaxis to reduce nosocomial SARS-CoV-2 transmission
Source: PLoS Comput Biol. 2025 Aug 5;21(8):e1013361. doi: 10.1371/journal.pcbi.1013361 (PMC12370187; doi:10.1371/journal.pcbi.1013361)
Supplement: S1 Fig — Individuals are described using an SEIR model (Susecptible, Exposed, Infected, Recovered). Patients infect each other directly via transmission (P2P direct), each other indirectly via contamination of the environment (P2P indirect), and healthcare workers (P2H). Healthcare workers (HCWs) are infected in the community independent of the hospital, and infect patients (H2P), and each other in hospital (H2H). (PDF) [file pcbi.1013361.s005.pdf]

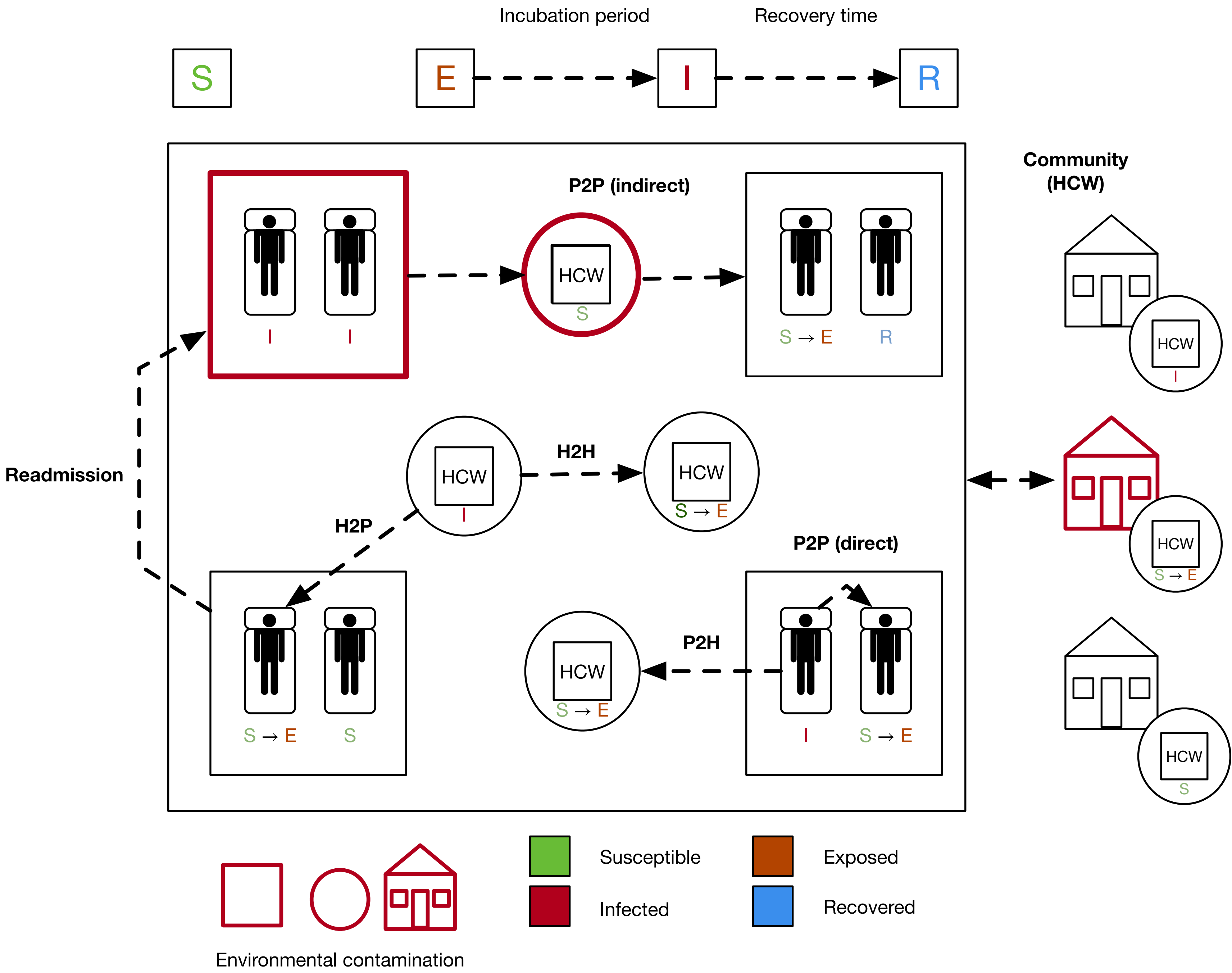
Incubation period
Recovery time
S
E
I
R
P2P (indirect)
HCW
S
I
I
S → E
R
Community
(HCW)
HCW
I
Readmission
HCW
I
H2H
HCW
S → E
H2P
HCW
S → E
P2P (direct)
S → E
S
P2H
HCW
S → E
I
S → E
HCW
S
Environmental contamination
Susceptible
Exposed
Infected
Recovered
