# Supplementary figures and images for: Modelling the potential use of pre-exposure prophylaxis to reduce nosocomial SARS-CoV-2 transmission
Source: PLoS Comput Biol. 2025 Aug 5;21(8):e1013361. doi: 10.1371/journal.pcbi.1013361 (PMC12370187; doi:10.1371/journal.pcbi.1013361)

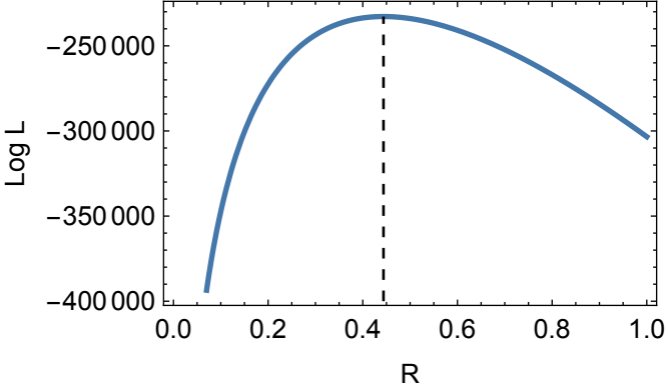

Supplement: S2 Fig — This calculation was performed on identified clusters of transmission between individuals from the original model, using the sizes of clusters to infer a value. We note that transmission is limited by the nature of the hospital environment, and by individuals not remaining in hospital for the duration of their infection. In this sense the estimate cannot be compared directly to estimates of R0 for general SARS-CoV-2 transmission. (PDF) [file pcbi.1013361.s006.pdf]

**A**

ACE2 expression: Model uncertainty

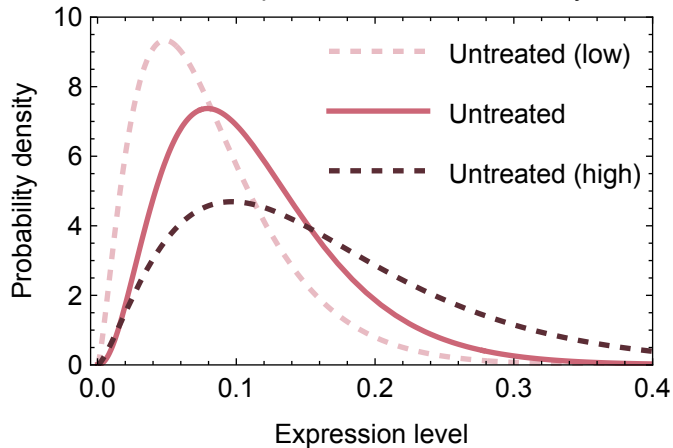**B**

ACE2 expression: Model uncertainty

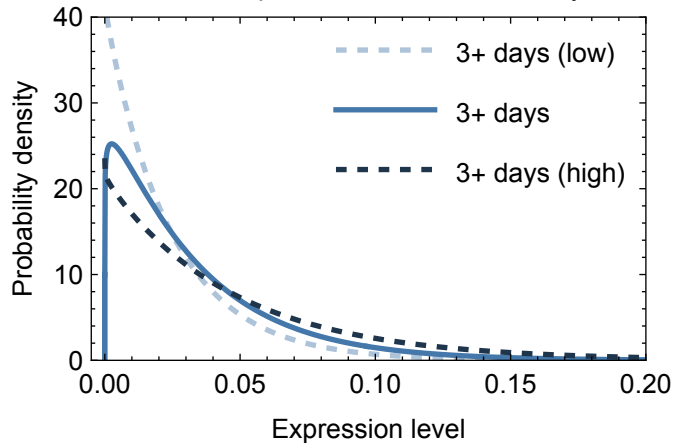

Supplement: S3 Fig — Solid lines show the original fits of a model to data describing ACE2 expression levels. Dashed lines show low (reduced mean expression) and high (increased mean expression) fits, differing by one log likelihood unit from the best fitting model. Data are shown. A. For untreated individuals. B. For treated individuals. A model in which the effect of UDCA was reduced was constructed by combining the low mean untreated case with the high mean 3 + days case. Likewise a model in which the effect of UDCA was increased was constructed by combining the high mean untreated case with the low mean 3 + days case. (PDF) [file pcbi.1013361.s007.pdf]

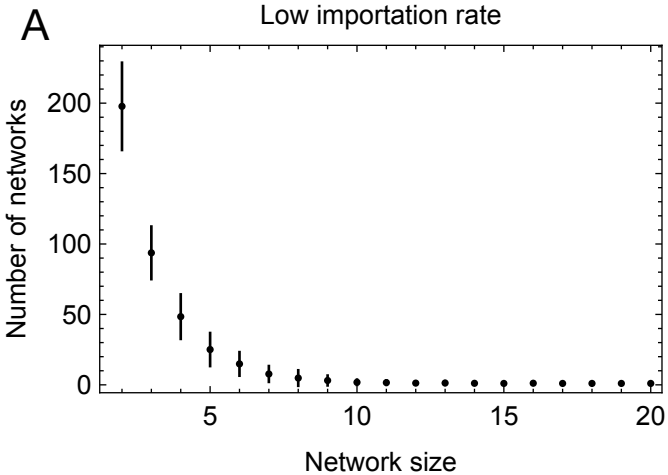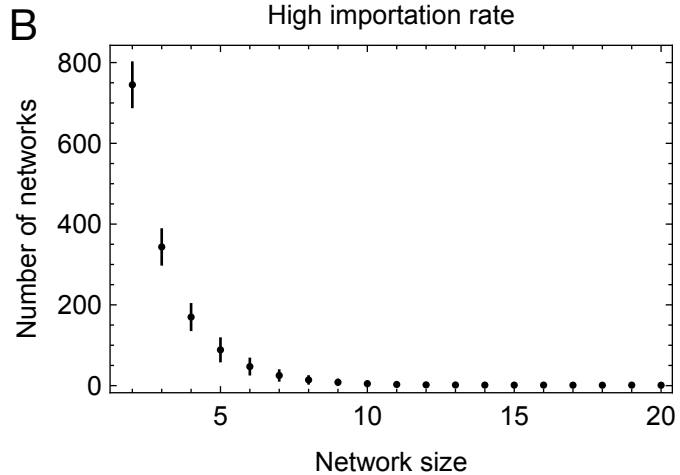

Supplement: S4 Fig — A cluster size of 2 indicates one person infecting another, with no further transmission. (PDF) [file pcbi.1013361.s008.pdf]

A

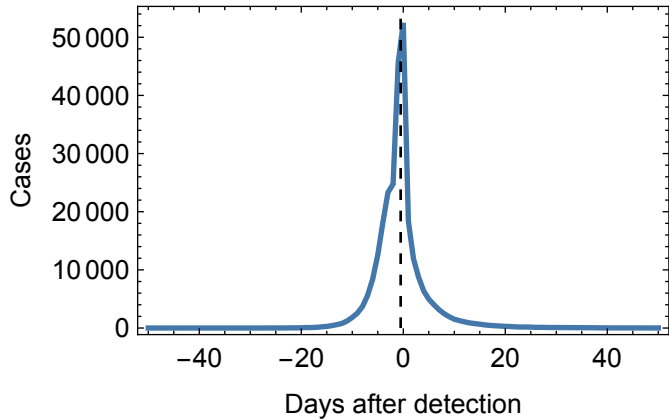

B

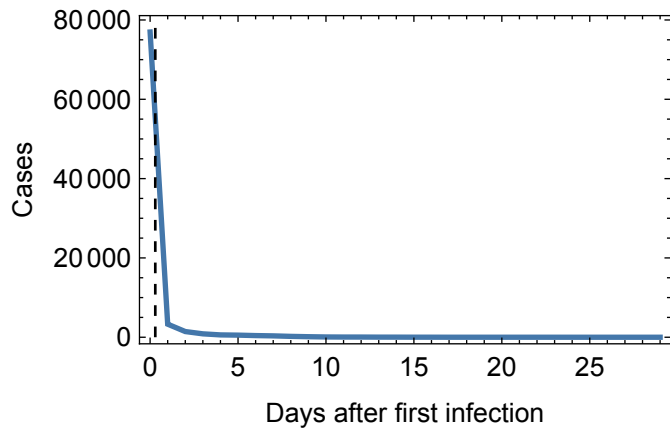

Supplement: S5 Fig — A. Distribution of days of infection of cases in clusters relative to the day on which the cluster was detected. The vertical dashed line shows the mean value: the mean date of infection was 0.51 days before the detection of the first case in a cluster. Data are shown for detected clusters. B. Infection times for undetected clusters of infection relative to the first time of infection within a cluster. The vertical dashed line shows the mean value: the mean date of infection was 0.29 days after the first infection in the cluster. (PDF) [file pcbi.1013361.s009.pdf]

**A**

90% high ACE2 expression

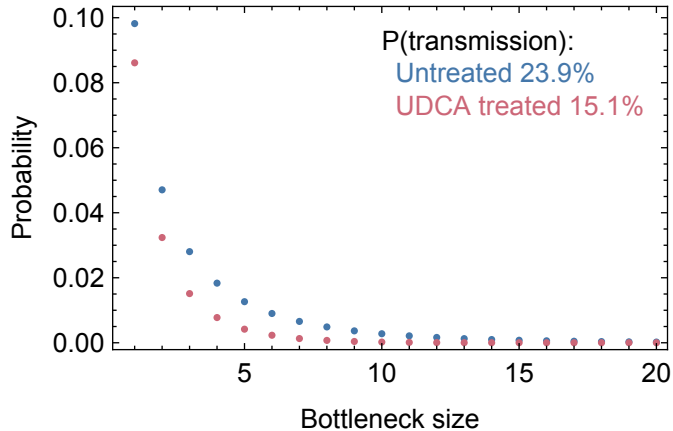**B**

10% low ACE2 expression

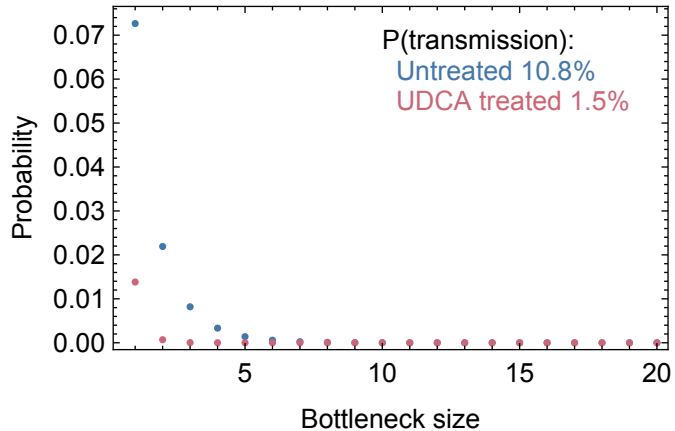

Supplement: S6 Fig — Inferred values are shown for among individuals at the 10th and 90th centiles of ACE2 expression, in untreated individuals, and in individuals treated for more than three days with UDCA. In both cases UDCA reduces expected bottleneck sizes, but with a more dramatic reduction in the probability of transmission (i.e., bottleneck size ≥ 1) among individuals with low ACE2 expression. (PDF) [file pcbi.1013361.s010.pdf]

Lythgoe et al 2021: SARS-CoV-2 data

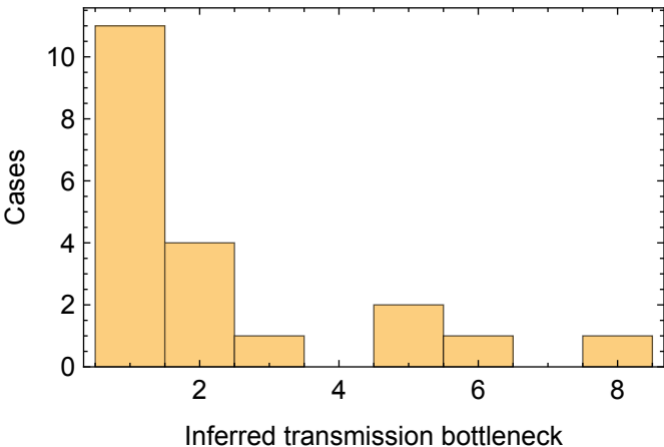

Supplement: S7 Fig — Data describe the number of SARS-CoV-2 viruses initiating infection in 20 cases of household transmission. In our study these data were augmented with cases describing non-infection, that is with transmission bottleneck zero, reflecting a published secondary attack rate for SARS-CoV-2 in a domestic context [35]. Our basic exposure model, described in Fig 1A, was then fitted to the augmented data. (PDF) [file pcbi.1013361.s011.pdf]

**A**

Discrete ACE2 expression values

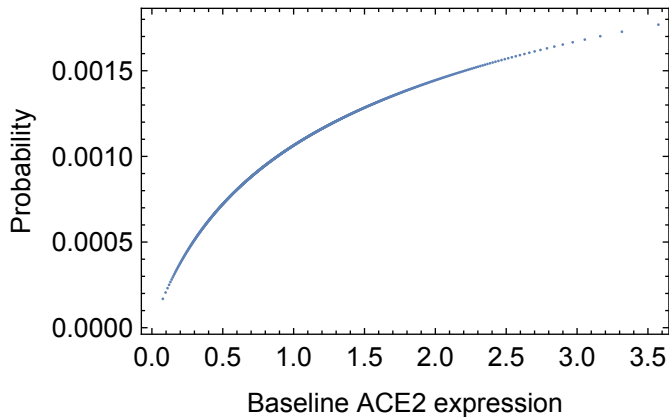**B**

Implied probability density function

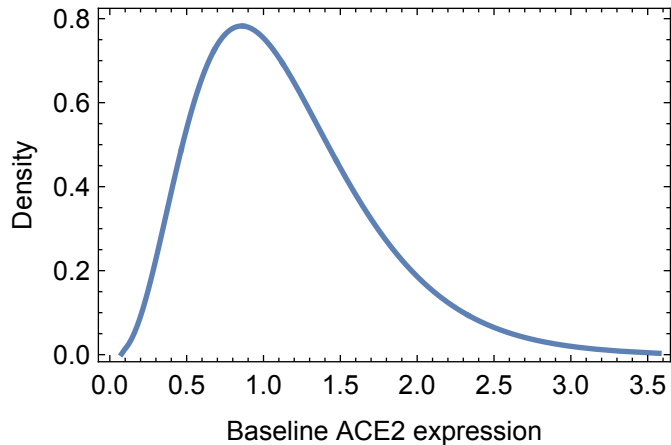

Supplement: S8 Fig — A. Probabilities of discrete values of baseline ACE2 expression. B. Probability density function implied by the discrete distribution. (PDF) [file pcbi.1013361.s012.pdf]
